# Polymorphism of SERPINE2 gene is associated with pulmonary emphysema in consecutive autopsy cases

**DOI:** 10.1186/1471-2350-11-159

**Published:** 2010-11-10

**Authors:** Koichi Fujimoto, Shinobu Ikeda, Tomio Arai, Noriko Tanaka, Toshio Kumasaka, Takeo Ishii, Kozui Kida, Masaaki Muramatsu, Motoji Sawabe

**Affiliations:** 1 Department of Molecular Epidemiology, Medical Research Institute, Tokyo Medical and Dental University, 2-3-10 Kanda-surugadai, Chiyoda-ku, Tokyo 101-0062, Japan; 2 Department of Pathology, Tokyo Metropolitan Geriatric Hospital, 35-2 Sakae-cho, Itabashi-ku, Tokyo 173-0015, Japan; 3 Department of Biostatistics, Harvard School of Public Health, Boston, MA, USA; 4 Department of Pathology, Juntendo University School of Medicine, 2-1-1 Hongo, Bunkyo-ku, Tokyo 113-8421, Japan; 5 Department of Pulmonary Medicine, Infection and Oncology, Nippon Medical School, 1-1-5 Sendagi, Bunkyo-ku, Tokyo 113-8602, Japan

## Abstract

**Background:**

The *SERPINA1*, *SERPINA3*, and *SERPINE2 *genes, which encode antiproteases, have been proposed to be susceptible genes for of chronic obstructive pulmonary disease (COPD) and related phenotypes. Whether they are associated with emphysema is not known.

**Methods:**

Twelve previously reported single nucleotide polymorphisms (SNPs) in *SERPINA1 *(rs8004738, rs17751769, rs709932, rs11832, rs1303, rs28929474, and rs17580), *SERPINA3 *(rs4934, rs17473, and rs1800463), and *SERPINE2 *(rs840088 and rs975278) were genotyped in samples obtained from 1,335 consecutive autopsies of elderly Japanese people. The association between these SNPs and the severity of emphysema, as assessed using macroscopic scores, was determined.

**Results:**

Emphysema of more than moderate degree was detected in 189 subjects (14.1%) and showed a significant gender difference (males, 20.5% and females, 7.0%; p < 0.0001). Among the 12 examined SNPs, only rs975278 in the *SERPINE2 *gene was positively associated with emphysema. Unlike the major alleles, homozygous minor alleles of rs975278 were associated with emphysema (odds ratio (OR) = 1.54; 95% confidence interval (CI) = 1.02-2.30; p = 0.037) and the association was very prominent in smokers (OR = 2.02; 95% CI = 1.29-3.15; p = 0.002).

**Conclusions:**

*SERPINE2 *may be a risk factor for the development of emphysema and its association with emphysema may be stronger in smokers.

## Background

The pathogenesis of chronic obstructive pulmonary disease (COPD) involves both small airway remodeling and emphysema [1]. Pulmonary emphysema is defined through pathological criteria as an "abnormal permanent enlargement of the airspaces distal to the terminal bronchioles, accompanied by destruction of the alveolar walls, and without obvious fibrosis" [2]. Emphysema progression is inextricably linked to the destruction of alveolar elastic fibers by elastolytic proteases associated with a chronic tobacco smoke-induced inflammatory process and to an antiprotease deficiency such as in alpha 1- antitrypsin deficiency [3].

Cigarette smoking may induce inflammation in the lungs and increase the release of proteases, which are counteracted by antiproteases in order to prevent parenchymal injury. However, smokers in whom COPD develops, the production of antiproteases may be inadequate to neutralize the effects of multiple proteases, possibly because of genetic polymorphisms that impair the function or production of these proteins [4]. Epidemiologic data indicate that over 95% of patients with COPD are current or former smokers; however, only about 10-20% of smokers develop COPD [5,6]. In this regard, additional factors such as genetic variability contribute to smoke susceptibility [7].

Serpins constitute a group of protease inhibitors comprising 36 human proteins [8]. Serpins regulate processes such as coagulation and inflammation by blocking targets of chymotrypsin-like serine proteases such as thrombin, trypsin, and human neutrophil elastase. As per the established nomenclature for serpins [9], the human genome encodes 16 clades of serpins--Serpin A through Serpin P--and the corresponding genes are further categorized into 29 inhibitory and 7 non-inhibitory genes [10]. *SERPINA1*, *SERPINA3*, and *SERPINE2 *are reported to be associated with COPD [11-22].

Alpha 1-antitrypsin deficiency is a high risk factor for COPD [11,12] and is associated with rare polymorphisms in the *SERPINA1 *gene. In fact, studies on common *SERPINA1 *variants have revealed that *SERPINA1 *haplotypes are associated with COPD [13], albeit not very conspicuously.

Alpha 1-antichymotrypsin, encoded by *SERPINA3*, is a plasma protease inhibitor that targets neutrophil cathepsin G and elastase [14]. In several cases, mutations in *SERPINA3 *are found to be associated with COPD [15]. A similar but less prominent association is found between COPD and common variants of *SERPINA3 *[16-18].

*SERPINE2 *encodes protease nexin 1--the archetypal member of the serine protease inhibitor family. DeMeo and associates first suggested that *SERPINE2 *is a COPD susceptibility gene and is probably influenced by a gene-by-smoking interaction in the Boston Early-Onset COPD study [19]. Subsequently, this was further validated and supported by additional data [20-22].

Although a number of large population studies are reported for COPD, few studies are well reproduced, probably due to the heterogenous mixture of COPD phenotypes. As COPD is composed of at least two components, the examination of emphysema might reduce the heterogeneity, and provide insight into specific mechanisms associated with mortality. In this study, we investigated 12 putative causal SNPs in *SERPINA1*, *SERPINA3*, and *SERPINE2 *by using autopsy samples obtained from 1,335 consecutive Japanese individuals. We then studied the association between these SNPs and emphysema, which was categorized on the basis of macroscopic scores.

## Methods

### Subject population

From February, 1995 to July, 2003, 1,536 autopsies were performed at the Department of Pathology, Tokyo Metropolitan Geriatric Medical Center, Tokyo, Japan. Of these, 1,335 (717 males and 618 females) were performed on Japanese cadavers, and the pathological findings of the lungs obtained during these autopsies were used in this study. The characteristics of the study population are available at an Internet-based database called "The Japanese SNP database for geriatric research (JG-SNP)" http://www.tmghig.jp/jg-snp/english/E_top.html.

Smoking status of the individuals was retrospectively determined by reviewing medical records, and subjects were classified as smokers (including ex-smokers) and life-time never-smokers. No medico-legal cases were included in the study. We did not use data from lung-function tests for association analysis because limited data were available, and because the duration between the conduction of these tests and death varied widely.

### Pathological assessment of emphysema

Both excised lungs were inflated with a 10% formalin solution at a constant pressure of 25 cm H_2_O. The lungs were section in the sagittal plane to obtained 2-cm-thick slices.

The type and severity of the emphysematous changes in all the lung slices were macroscopically assessed by a pulmonary pathologist (K.T.). Emphysema was classified as centrilobular emphysema (CE), panlobular emphysema (PE), or focal emphysema (FE), and its severity was scored on a scale of 0-4 for each slice, by using a previously described method [2,23], where 0 = no changes, 1 = minimal changes, 2 = mild changes, 3 = moderate changes, and 4 = severe changes. The mean scores for slices from both lungs were used in an association analysis of single nucleotide polymorphisms (SNPs) (*see below*). In this study, emphysema was defined as CE changes with a score of >2 and/or PE changes with a score of ≥ 1, since CE changes are known to be mainly caused by smoking, and PE with CE is considered an extension of severe CE [24]. Patients whose emphysema scores for both lungs differed by more than 2 points were excluded from the study, because in general, the bilateral severity of the emphysema is closely correlated [24], and such wide differences might have been due to additional unusual effects.

### SNP selection

We selected 7 *SERPINA1 *SNPs (rs8004738, rs17751769, rs709932, rs11832, rs1303, rs28929474, and rs17580), 3 *SERPINA3 *SNPs (rs4934, rs17473, and rs1800463), and 2 *SERPINE2 *SNPs (rs840088 and rs975278) by referring to previous studies on COPD phenotypes [13,15,16,19,20]. The SNPs whose minor allele frequencies (MAFs) in the Japanese population were found to be less than 0.1 in the HapMap project http://hapmap.ncbi.nlm.nih.gov/ were excluded from this study. One of the *SERPINE2 *SNPs (rs840088; intron 1) has been reported to be associated with COPD in both family and case-control studies [19], and the other (rs975278; intron 5) has been reported to be associated with COPD phenotypes in 2 large populations [20]. We also screened for the PiS variant (rs17580) and PiZ variant (rs28929474), which are known to be the most common variants associated with alpha 1-antitrypsin deficiency [25,26].

### Genotyping

Genomic DNA was extracted from the renal cortex by the phenol-chloroform method and stored at -20°C until use. For genotype analysis, 10-ng aliquots of DNA were introduced into 384-well polymerase chain reaction (PCR) plates by using an automated fluid dispenser (BioMek2000; Beckman Coulter Inc., Fullerton, CA) and dried in a dry chamber.

Two *SERPINE2 *SNPs (rs840088 and rs975278) and one *SERPINA1 *SNP (rs1303) were genotyped using Taqman 5'-exonuclease assays, with primers obtained from Applied Biosystems Inc. (Foster City, CA). Major and minor allele probes were labeled with different colored fluorophores, and the fluorescence signals were detected using the ABI Prism 7900HT sequence detector system. The remaining 9 SNPs were genotyped using a melting curve analysis on a LightCycler 480 system (Roche Diagnostics K. K., Tokyo, Japan). Pathological assessments and genotyping were performed at different facilities in a double-blind manner. The PCR primers and hybridization probes used for the melting curve analysis are listed in additional file 1, table S1.

### Ethical considerations

All the autopsies and subsequent medical and genetic studies were performed with the written informed consent of the bereaved family of each patient. The use of autopsy materials for medical education and research is generally permitted by the Act of Postmortem Examinations of Japan. This study was approved by the ethics committees of Tokyo Metropolitan Geriatric Medical Center and Tokyo Medical and Dental University.

### Statistical analysis

Allelic test and genotype test in recessive model were used to detect the genetic contributions. Statistical analysis was performed using SAS ver. 9.1.3 (SAS Institute Inc., Cary, NC). Multiple logistic regression analyses were performed to examine the emphysema-related differences in the patients' clinical backgrounds and to calculate the odds ratio (ORs) and 95% confidence intervals (CIs). The estimates were adjusted for gender, age at the time of death, and smoking status. The Hardy-Weinberg equilibrium for all SNPs was tested using the chi-square goodness-of-fit test. Haplotype analyses of 5 SNPs (rs8004738, rs17751769, rs709932, rs11832, and rs1303) were performed using the stochastic expectation-maximization (EM) algorithm, which is incorporated in the THESIAS program http://ecgene.net/genecanvas/uploads/THESIAS3.1/Documentation3.1.htm. A p value of < 0.05 was considered statistically significant. The p values have not been adjusted for multiple testing.

## Results

### Study participants

The baseline characteristics of the subjects are shown in Table 1. The mean age at the time of death was 80.1 years. Of the 1355 subjects, 99 (7.4%) had CE with minimal changes, 248 (18.6%) had mild changes, 156 patients (11.7%) had moderate changes, 28 patients (2.1%) had severe changes, and the remaining 804 patients (60.2%) had no detectable emphysema. The pathological diagnosis of emphysema, which was defined as previously described, was made on 14.2% of the subjects (n = 189). Emphysema was more common among men than among women (20.5% vs. 7%; p < 0.0001). Further, 47.1% of the study population had a history of smoking, with a significant gender difference (males vs. females; 69.6% vs. 21.0%; p < 0.0001). Smoking was found to be a significant risk factor for the development of emphysema in the total study population (OR = 4.45, 95% CI = 2.88-7.06; p < 0.0001), and posed a slightly higher risk in women (OR = 5.22, 95% CI = 2.59-10.70; p < 0.0001) than in men (OR = 4.14, 95% CI = 2.40-7.65; p < 0.0001).

**Table 1:** Clinical characteristics of the cases

	Total (%) N = 1335	Subjects with emphysema (%) N = 189	Subjects without emphysema (%) N = 1146	p values
Men (%)	717 (53.7%)	147 (77.8%)	570 (49.7%)	<0.0001*
Age (years)	80.1 ± 9.0	81.3 ± 7.3	79.9 ± 9.3	<0.0001^†^
History of smoking				
Smokers	629 (47.1%)	146 (77.2%)	483 (42.1%)	<0.0001^‡^
Non-smokers	610 (45.7%)	32 (16.9%)	578 (50.4%)	
Data not available	96 (7.2%)	11 (5.8%)	85 (7.4%)	
Pack-years	21.2 ± 36.3	45.0 ± 40.5	17.7 ± 31.9	<0.0001^‡^

*Multiple logistic regression analysis adjusted for age and smoking history.

†Multiple logistic regression analysis adjusted for sex and smoking history.

‡Multiple logistic regression analysis adjusted for age and sex.

### Association between genetic polymorphisms of candidate genes and emphysema

The distribution of the 12 SNP genotypes and their association with emphysema are shown in Table 2. Neither the PiZ and PiS variants (rs28929474 and rs17580 respectively) nor the 2 reportedly rare variants of *SERPINA3 *(rs17473 and rs1800463) were detected in any of the samples examined. The MAFs obtained in our analysis did not considerably deviate from the data obtained in the HapMap project http://hapmap.ncbi.nlm.nih.gov/. The distribution of all genotypes did not significantly deviate from the Hardy-Weinberg equilibrium.

**Table 2:** Association of 12 SNPs with emphysema

Gene and SNP	Allele	Genotypic frequency in subjects with emphysema (n = 189) MM/Mm/mm	Genotypic frequency in subjects without emphysema (n = 1146) MM/Mm/mm	p value for HWE	p value for minor allele carriers*	p value for minor allele homozygotes*
*SERPINA1*						
rs8004738	A/G	.26/.50/.24	.24/.51/.25	.873	.761	.459
rs17751769	A/G	.26/.50/.24	.24/.50/.26	.858	.902	.573
rs709932	G/A	.53/.40/.07	.56/.37/.07	.450	.439	.866
rs1303	A/C	.47/.42/.11	.51/.40/.09	.896	.275	.572
rs11832	A/G	.45/.42/.13	.49/.40/.11	.148	.217	.416
*SERPINA3*						
rs4934	A/G	.37/.44/.19	.35/.47/.18	.440	.699	.910
*SERPINE2*						
rs840088	C/T	.32/.49/.19	.35/.48/.17	.928	.699	.908
rs975278	A/G	.26/.47/.27	.29/.52/.19	.329	.175	**.037**

*Multiple logistic regression analysis adjusted for age, sex, and smoking history.

Statistically significant values (p < 0.05) are shown in boldface.

p values shown above have not been adjusted for multiple testing

Abbreviations: MM, major allele homozygote; Mm, heterozygote; mm, minor allele homozygote; HWE, Hardy-Weinberg equilibrium

Subjects with and without emphysema did not show any differences in the genotypic distribution of 5 *SERPINA1 *SNPs and 1 *SERPINA3 *SNP. However, significant differences were found in the genotypic distribution of 1 *SERPINE2 *SNP (rs975278). This SNP was significantly associated with emphysema (OR = 1.54, 95% CI = 1.02-2.30; p = 0.037)

### Association of the SERPINE2 SNP rs975278 and smoking with emphysema

We next examined whether the effects of rs975278 differed between smokers and non-smokers. Among the smokers, the allelic and genotypic frequencies of this SNP were significantly different between subjects with and without emphysema (Table 3). A high frequency of GG homozygosity was significantly associated with an increased OR for emphysema (OR = 2.02, 95% CI = 1.29-3.15; p = 0.002).

**Table 3:** Association of rs975278 with emphysema in smokers

Variables	Subjects with emphysema (n = 127)	Subjects without emphysema (n = 425)	p value*	Odds ratio (95%)*
Genotype				
GG	42 (33%)	83 (20%)	**.002**	**2.02 (1.29-3.15)**
AG+AA	85 (67%)	342 (80%)		
Allele				
G	0.57	0.45	**.001**	**2.61 (1.45-4.76)**
A	0.43	0.55		

*Multiple logistic regression analysis adjusted for age and sex.

Statistically significant figures (p < 0.05) are shown in boldface.

p values shown above have not been adjusted for multiple testing

We performed haplotype analyses for 5 adjacent *SERPINA1 *SNPs, but none of the haplotypes was found to be associated with emphysema (data not shown).

## Discussion

The present study revealed that a polymorphism in *SERPINE2 *(rs975278) was associated with emphysema in consecutive autopsies of Japanese subjects. In the total study population, the OR for minor allele homozygotes was 1.54 (95% CI = 1.02-2.30). Among smokers, the OR for minor allele homozygotes was 2.02 (95% CI = 1.29-3.15). If the p values are corrected with multiple testing of twelve SNPs, the data without smoking stratification does not show any statistical significance. Among the smokers, however, the differences of the allelic and genotypic frequencies between subjects with and without emphysema were significant (p value × 12 < 0.05). To our knowledge, this is the first study to report that a *SERPINE2 *variant is closely associated with emphysema, especially in smokers.

### SERPINE2

*SERPINE2 *encodes a 44-kDa cellular and extracellular matrix-associated serine protease inhibitor [27], which is expressed in the developing and adult lung as well as in the airway epithelial cells [19]. The SERPINE2 protein mainly functions in coagulation and fibrinolysis by inhibiting thrombin, urokinase, and plasmin [27,28]. Plasmin is capable of contributing to metalloproteinase activation [29], thus, *SERPINE2 *may play an important role in lung physiology [30].

*SERPINE2 *was initially identified as a positional candidate for COPD and related phenotypes on chromosome 2q33-35 [31]. Subsequently the association of SNPs in *SERPINE2 *with COPD was observed in a case-control study in smokers [19]. Three independent groups attempted to replicate the findings of the above study: one study successfully replicated the association [20], while the others did not [32,33]. Further, *SERPINE2 *polymorphisms were used to study COPD-related phenotypes; association with hypoxemia with severe COPD [34] and with bronchodilator responsiveness in COPD [35] were observed. Thus, accumulating evidence shows that *SERPINE2 *is a susceptible gene of COPD and a modifier of COPD related phenotypes, but its relation with emphysema remains unknown. Our finding that *SERPINE2 *polymorphism is associated with emphysema appears to be consistent with the findings of these previous studies that reported positive associations [19,20], as assessed using physiological parameters of lung function. According to the Hapmap project, the allele frequencies of the polymorphism (rs975278) are different in population groups, however, the risk allele (G allele) pointed to the same direction in the present study. These previous findings were further explored in our study, which analyzed pathological findings in consecutive autopsy cases.

In this study, the association of *SERPINE2 *polymorphism with emphysema was more prominent among the smokers. This suggests that SERPINE2 variants contribute to the risk of developing emphysema in smokers. While the reason for this interaction is presently unclear, many smoking-associated genes have been identified [36]. It is tempting to speculate that SERPINE2 polymorphism may affect SERPIMI2 expression after exposure to cigarette smoke and may thus hamper the repair of smoking-induced lung damage. However, the mechanism by which SERPINE2 contributes to the development of emphysema remains to be elucidated.

We also examined whether the variants studied were associated with the typical complications related to COPD, including osteoporosis, aortic aneurysm, myocardial infarction, aortic dissection, cerebral infarction, intracranial hemorrhage, and interstitial pneumonitis. Our results in this regard were negative (data not shown).

### Emphysema and COPD

COPD is a clinical condition that is diagnosed by physiological tests, whereas pulmonary emphysema is diagnosed by pathological examinations. Although these conditions are not identical, both are indicative of a disease involving airway obstruction and chronic bronchitis. An important feature of our study is that we performed pathological examinations to evaluate the disease phenotype; such analysis is usually not possible in routine clinical practice. The genetic contribution we investigated for emphysema may be more reflecting the final steps of the COPD that involve severe alveolar damage due to imbalance of proteaseses and their inhibitors.

With the recent advancements in computed tomography (CT), high-resolution images that enable a reasonably accurate assessment of pulmonary emphysema can be obtained [37]. Emphysema appears to be correlated with the severity of lung function impairment in COPD [38]; however, attempts have been made to categorize COPD, regardless of whether emphysema is detected using CT. Some candidate gene polymorphisms are reported to be associated with emphysema, as diagnosed using CT [39-41]. We believe that *SERPINE2 *is a good candidate gene that is associated with emphysema, as diagnosed using CT.

Thus, our finding that a *SERPINE2 *variant is associated with emphysema may indicate that this variant influences the severity of lung destruction rather than the presence or absence of chronic pulmonary obstruction. Future studies should aim to elucidate the genetic background of COPD with and without pulmonary emphysema.

### Limitations

Spirometric data were not sufficiently available in this study since the samples were obtained during autopsies of elderly subjects. Small number of subjects, especially only 32 non-smoker patients have pulmonary emphysema, may reduce power to detect genetic associations. The present study included only those individuals who underwent autopsy and had an average age with emphysema was similar to those without emphysema (Table 1). This implies that the study population may have been skewed towards individuals with mild disease, and that a survival bias may have been present. The elderly population is likely to be exposed to long-term environmental effects other than smoking; however, the present study did not consider these effects.

## Conclusions

The present study revealed that a *SERPINE2 *SNP (rs975278) was associated with pathologically diagnosed emphysema in consecutive autopsies of Japanese smokers. Further studies are required to clarify the molecular and biochemical mechanisms underlying this association.

## Competing interests

This study was supported in part by a grant from the Smoking Research Foundation to M.S., Tokyo, Japan.

## Authors' contributions

SI performed the SNP genotyping; TA prepared the clinicopathological data; NT conducted the statistical analysis; TK assessed the lung pathology; TI and KK reviewed the manuscript; MM provided general instructions on SNP genotyping and manuscript writing; and MS prepared the clinicopathological data and provided general instructions on the study. All authors read and approved the final manuscript.

## Pre-publication history

The pre-publication history for this paper can be accessed here:

http://www.biomedcentral.com/1471-2350/11/159/prepub

## Supplementary Material

Additional table S1 **Primers and probes used for genotyping**. The PCR primers and hybridization probes used for the melting curve analysis. Click here for file
