# Supplementary material for: Polymorphism of SERPINE2 gene is associated with pulmonary emphysema in consecutive autopsy cases
Source: BMC Med Genet. 2010 Nov 10;11:159. doi: 10.1186/1471-2350-11-159 (PMC2994839; doi:10.1186/1471-2350-11-159)
Supplement: Additional table S1 — Primers and probes used for genotyping. The PCR primers and hybridization probes used for the melting curve analysis. [file 1471-2350-11-159-S1.DOC]

| Target  polymorphisms | Forward primer | Reverse primer | Anchor probe | Detection probe |
| --- | --- | --- | --- | --- |
| rs4934 | TCTGGCCCTCTGAGACTTAAA | TGGGTCAGATTCTCCTCG | GGGCAGAACCCAGCCGCCA-Fluorescein | LC Red 640-GAGCCCCAGAGCCAG-Phosphate |
| rs11832 | CCCTCCGTCTCTACCAG | CTTGTGTGGGAAACAGTCG | TCCTAGATTAGTTTGGGATGGGGCAGTGCCT-Fluorescein | LC Red 640-CCACAGGACAAGGCCA-Phosphate |
| rs17473 | TCCATCTTGTCTTGATCAGGG | TGAGCAAGAAAAAGTGGGTAAT | LC Red 640-TCAGGTGATGCAAACTCATCATGGGCACC-Phosphate | CTCGTCCCGGAAGTAAGCTATA-Fluorescein |
| rs709932 | TCAACCTCACGGAGATTCC | CCAAAAACTTATCCACTAGCTT | LC Red 640-AACCAGCCAGACAGCCAGCTCC-Phosphate | GGAACTCCTCCGTACCCT-Fluorescein |
| rs1800463 | GTACAAGCAGTTAGTCCTGAAG | CTGGAAGCTCTGGTGAATTT | LC Red 640-CCACCGCCTTGGCCTTCCTGT-Phosphate | CTCCCCACCGAGCATC-Fluorescein |
| rs8004738 | TGGGTGGGCAGGAACTG | CCCCTCCAACCTGGAAT | LC Red 640-CTAGCCGCTGCTGCTG-Phosphate | GGGTCGGGCCTCCGAGGAAGG-Fluorescein |
| rs17751769 | CTGGCTGAATGGATATTCCG | GACAGCAGGGCTTAGAGT | GAGAGGGGAAGAAATGCCCAGGAGCTACCG-Fluorescein | LC Red 640-GGGCAGGCGACCTCA-Phosphate |
| rs17580 | GGTGCCTATGATGAAGCGTTTAGGC | AGGTGTGGGCAGCTTCTTGGTCA | TTCTTCCTGCCTGATGAGGGGAAACTA-Fluorescein | LC Red 640-GCACCTGGAAAATGAAC-Phosphate |
| rs28929474 | GGTGTCCACGTGAGCCTTGC | AAAAACATGGCCCCAGCAGCT | CTCCAGGCCGTGCATAAGGCTGT-Fluorescein | LC Red 640-GACCATCGACGAGAAAGGG-Phosphate |

**Additional Table S1**. Primers and probes used for genotyping
